# Integration and Comparison of Transcriptomic and Proteomic Data for Meningioma

**DOI:** 10.3390/cancers12113270

**Published:** 2020-11-05

**Authors:** Jemma Dunn, Vasileios P. Lenis, David A. Hilton, Rolf Warta, Christel Herold-Mende, C. Oliver Hanemann, Matthias E. Futschik

**Affiliations:** 1 Faculty of Health: Medicine, Dentistry and Human Sciences, The Institute of Translational and Stratified Medicine, University of Plymouth, The John Bull Building, Plymouth Science Park, Research Way, Plymouth PL6 8BU, UK; jemma.dunn@plymouth.ac.uk; 2 School of Health & Life Sciences, Centuria Building, Teesside University, Middlesbrough, Tees Valley TS1 3BX, UK; V.Lenis@tees.ac.uk; 3 Cellular and Anatomical Pathology, Plymouth Hospitals NHS Trust, Derriford Road, Plymouth PL6 8BU, UK; davidhilton@nhs.net; 4 Department of Neurosurgery, Division of Experimental Neurosurgery, Heidelberg University Hospital, 69120 Heidelberg, Germany; Rolf.Warta@med.uni-heidelberg.de (R.W.); Christel.Herold-Mende@med.uni-heidelberg.de (C.H.-M.); 5 Faculty of Medicine, School of Public Health, Imperial College London, Medical School, St Mary’s Hospital, Praed Street, London W2 1NY, UK

**Keywords:** meningioma, transcriptomics, proteomics, data integration, biomarker, drug targets, MAOB

## Abstract

**Simple Summary:**

Meningioma are the most common primary intracranial tumour, yet gaps in their molecular characterisation persist and effective treatments for aggressive, recurring, high-grade (WHO grade III) meningioma are lacking. The aim of our study was to outline differences in the molecular landscape between high-grade and low-grade (WHO grade I) meningioma by combining transcriptomic and proteomic data from two former independent studies. We described both concordant and discordant differential expression between grades and validated expression at either the transcript or protein level for genes including *MAOB, CST3*, *LAMP2*, *PACS1* and *HTRA1*. Concordantly upregulated transcripts/proteins of high-grade tumours were enriched in biological processes such as oxidative phosphorylation and RNA metabolism. Using an integrated omics approach we have provided a previously unreported, valuable insight into the altered transcript/protein expression profiles between high and low-grade meningioma. In addition, our findings identified molecules that may hold potential as biomarkers or therapeutic targets of high-grade meningioma.

**Abstract:**

Meningioma are the most frequent primary intracranial tumour. Management of aggressive meningioma is complex, and development of effective biomarkers or pharmacological interventions is hampered by an incomplete knowledge of molecular landscape. Here, we present an integrated analysis of two complementary omics studies to investigate alterations in the “transcriptome–proteome” profile of high-grade (III) compared to low-grade (I) meningiomas. We identified 3598 common transcripts/proteins and revealed concordant up- and downregulation in grade III vs. grade I meningiomas. Concordantly upregulated genes included *FABP7*, a fatty acid binding protein and the monoamine oxidase *MAOB*, the latter of which we validated at the protein level and established an association with Food and Drug Administration (FDA)-approved drugs. Notably, we derived a plasma signature of 21 discordantly expressed genes showing positive changes in protein but negative in transcript levels of high-grade meningiomas, including the validated genes *CST3*, *LAMP2*, *PACS1* and *HTRA1*, suggesting the acquisition of these proteins by tumour from plasma. Aggressive meningiomas were enriched in processes such as oxidative phosphorylation and RNA metabolism, whilst concordantly downregulated genes were related to reduced cellular adhesion. Overall, our study provides the first transcriptome–proteome characterisation of meningioma, identifying several novel and previously described transcripts/proteins with potential grade III biomarker and therapeutic significance.

## 1. Introduction

Meningioma arise from the meninges, the tissue layers that envelop the brain and spinal cord, and account for approximately 36% of all primary central nervous system (CNS) tumours [1]. According to the current WHO classification, around 80% of meningiomas are benign (grade I), 20–25% atypical (grade II) and 1–6% malignant (grade III) [1]. Standard treatment is surgical resection and can be curative [2]. However, excision is not without risk of postoperative morbidity, and, dependent upon location, tumours may be incompletely resected or surgically inaccessible [2]. Here, radiotherapy is recommended as either adjunct or monotherapy, whilst chemotherapy is ineffective in meningioma [3]. Notably, therapeutic management following surgical removal of WHO grade III cranial meningioma remains challenging, with a 5-year survival of just 30% in comparison to 80% for WHO grade II and 90% for WHO grade I tumours [4]. Furthermore, *de novo* WHO grade III anaplastic meningiomas have been found to be associated with a dismal mean overall survival of 3.1 years [5]. 

Recent studies have started to unravel the molecular landscape of meningioma. In addition to the established association of meningioma with pathogenic aberrations in the tumour suppressor gene *NF2*, genomic analyses have identified genes including *TRAF7*, *KLF4*, *AKT1*, *SMO*, *PIK3CA* and *POLR2A* to possess recurrent mutations in low grade non-*NF2* mutant meningioma [6,7,8,9]. Specific to grade III meningioma, somatic driver mutations in the SWI/SNF chromatic regulatory complex, most frequently in the SWI/SNF component AIRD1A, have recently been revealed and linked to poor prognosis [10]. Transcriptomic profiling of meningioma grades has further facilitated the discovery of candidate genes displaying prognostic potential [11,12,13,14,15]. Notably, Schmidt et al. (2016) performed a comparative transcriptomic analysis across all grades comprising the largest cohort of rare grade III meningioma to date [12]. Although the study provided comprehensive coverage of the transcriptional meningioma landscape and identified PTTG1 and LEPR as prognostic markers of aggressive behaviour, it remained to be demonstrated whether the transcriptional changes observed were generally indicative of changes in protein abundance. Since proteins exert the intended function of a majority of genes, it is important to assess the predictive value of an altered transcriptomic profile at the protein level. Moreover, proteins are subject to many post-translational modifications undetectable at the transcript level, such as phosphorylation, which regulate protein structure, functional state and subcellular compartmental localisation. Previous integrations of transcriptomic and proteomic profiles generated from mammalian cell lines, including some cancer cell lines, have shown a general overall correlation of transcript and protein levels despite notable differences [16,17,18].

Proteomic profiling studies in meningioma have enabled the grade-wise comparison of differential expression patterns as well as the identification of promising biomarkers or therapeutic targets [19,20]. However, it has become apparent that the integration of different omics approaches is necessary to gain a more complete picture of the molecular alterations underlying a complex pathology than the analysis of individual omics datasets can provide [21]. Indeed, several recent studies have utilised multiple omics platforms with varying levels of data integration to reveal a comprehensive molecular understanding of meningioma pathogenesis. Sahm et al. (2017) described a DNA methylation-based meningioma classification system demonstrating greater improved accuracy for predicting recurrence and prognosis than the WHO grade alone and went on to characterise the system by DNA copy number analysis, mutational profiling and RNA sequencing [22]. Interestingly, grade III meningioma mapped into two distinct methylation classes associated with differing progression-free survival outcomes [22]. Additional studies have identified further molecular signatures attributed with high-grade meningioma by integration of genomic analyses, including upregulation of the FOXM1 transcription factor network, an upregulation of *HOX* genes and mutations of the SWI/SNF chromatin regulatory complex [10,23,24,25]. Nevertheless, a proteomic dataset has yet to be incorporated into a multi-omics approach. 

In the present study, we integrated two former independent but comparable meningioma omics datasets covering the transcriptome and proteome to compare the molecular states of grade I and grade III meningioma [12,19]. Both datasets stand out as providing the largest cover of grade III meningioma to date. We identified coherent patterns in transcriptomic and proteomic profiles, revealed common functional trends in the combined datasets and elucidated upon underlying causes of discordant expression between datasets. Our findings might not only assist in the interpretation of transcriptomic studies and boost their predictive power but also provide clues about the type of dysregulated expression observed in meningioma. 

## 2. Results

### 2.1. Integrated Transcriptome–Proteome Analysis of Grade III vs. Grade I Meningioma

**Figure 1:**
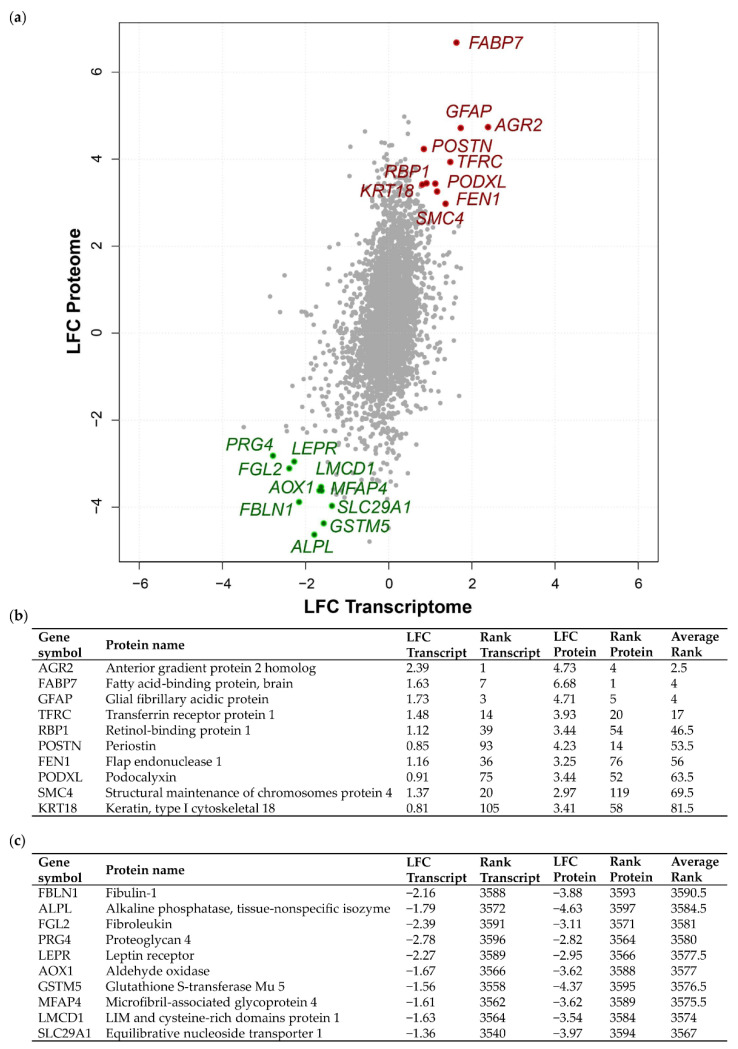
Concordant transcriptome–proteome differential expression of grade III vs. grade I meningiomas. (**a**) Scatterplot displaying 3598 genes commonly identified in a transcriptomic microarray analysis including 13 non-recurrent grade I meningiomas and 20 malignant grade III meningiomas and a global proteomic analysis including eight grade I and six grade III meningiomas [12,19]. Pearson’s correlation coefficient revealed a modest, yet highly significant correlation of *r* = 0.35, *p* = 3.6 × 10^−104^. Log_2_ fold changes (LFC) at the transcript and protein level of overlapping genes are shown for grade III vs. grade I meningiomas. The top 10 genes based on average rank value exhibiting concordantly increased or decreased LFC between the two datasets are highlighted in red or green, respectively. (**b**,**c**) Top 10 genes showing concordantly increased (**b**) or decreased (**c**) LFC in grade III vs. grade I meningiomas are presented with respective LFC, rank and average rank values. Table S3A provides details of all 3598 genes common to both analyses, transcript/protein LFC, their rank and average rank.

To obtain a more comprehensive view of the molecular changes in meningiomas of different grades, we integrated previously published proteomic measurements [19] with an extended meningioma transcriptomic dataset [12]. Here, we focused on comparing the transcriptome–proteome of grade III vs. grade I meningioma in order to dissect the altered molecular signature of the most aggressive meningioma subgroup. Additionally, the integration allowed us to investigate concordant as well as discordant alterations within the transcriptome–proteome dataset. We detected 3598 genes common to both omics datasets following the mapping of transcripts and proteins to Entrez Gene IDs. For the shared genes, we calculated grade III vs. grade I meningioma log_2_ fold changes (LFC) (Figure 1a; Table S3A). Comparison of the transcriptomic and proteomic LFC distributions showed notable differences in spread and mean (Figure S1). Proteins displayed a greater LFC range and a mean of 0.41 while transcript LFCs exhibited a narrower range with a mean of 0.0. The divergence observed here could be due to differences in underlying data processing and normalisation methods. Despite these differences, mRNA and protein levels displayed a highly significant positive Pearson’s correlation coefficient of 0.35 (*p* = 3.6 × 10^−104^ Figure 1a). Nevertheless, the difference observed in dataset distributions warranted caution indicating that direct integration of datasets based on original LFC values would not be suitable and may lead to systematic biases. Therefore, we employed a rank-based integration approach that is robust against the observed variation in LFC distribution (Table S3A). This approach enabled the definition of concordant and discordant expression of the grade III meningioma transcriptome–proteome (see Materials and Methods). The top 10 genes showing increased or decreased concordant differential expression between grade III vs. grade I meningioma at both transcript and protein level are highlighted in Figure 1a. Further, assigned transcript/protein rank and average rank value of the top 10 concordantly upregulated or downregulated genes are presented in Figure 1b,c. Among the top genes with concordant upregulated expression was the known oncogene *AGR2* with the lowest average rank of 2.5, a transcript LFC of 2.39 and protein LFC of 4.73, as well as *FABP7,* which had an average rank of 4, a transcript LFC of 1.63 and protein LFC of 6.68 (Figure 1b). Concordantly downregulated genes included FBLN1, which encodes the extracellular glycoprotein fibulin-1 (FLBN1), with the highest average rank of 3590.5, a transcript LFC of −2.16 and protein LFC of −3.88. We also detected the leptin receptor, LEPR, to be downregulated in grade III meningioma, consistent with previous findings of its reduced abundance in higher grade compared to low-grade meningiomas by Schmidt et al. (2016) and Menghi et al. (2011) [12,26]. Further, concordantly downregulated genes included *MFAP4*, a member of the microfibrillar-associated protein family, MFAP, which presented an average rank of 3575.5, a transcript LFC of −1.61 and protein LFC of −3.62 (Figure 1c). 

**Figure 2:**
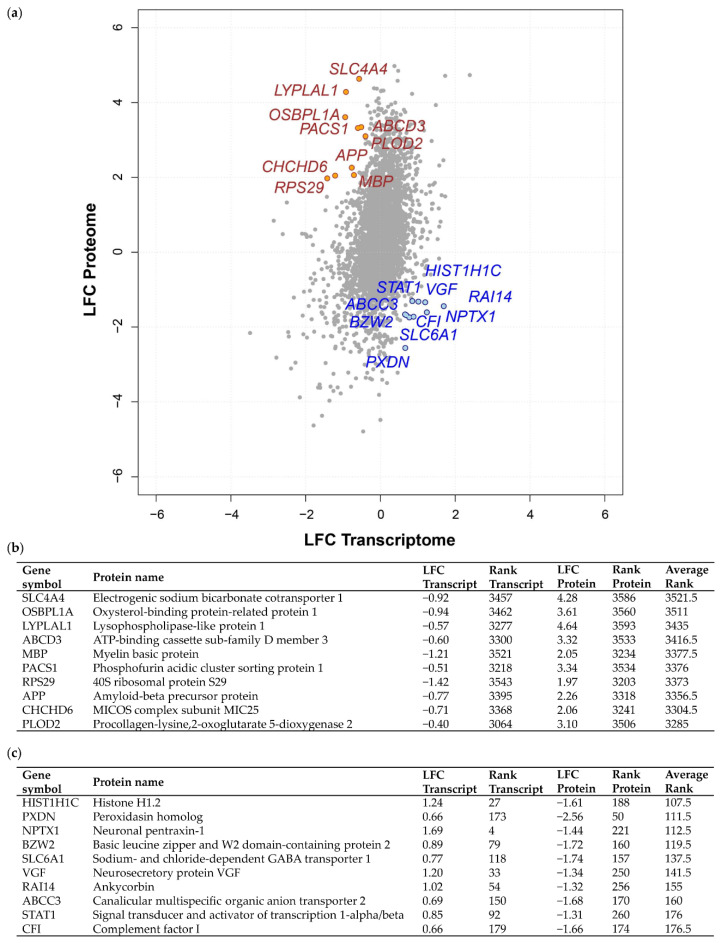
Discordant transcriptome–proteome differential expression of grade III vs. grade I meningiomas. (**a**) Scatterplot displaying 3598 genes commonly identified in a transcriptomic microarray analysis including 13 non-recurrent grade I meningiomas and 28 malignant grade III meningiomas and a global proteomic analysis including eight grade I and six grade III meningiomas [12,19]. Log_2_ fold changes (LFC) at the transcript and protein level of overlapping genes are shown for grade III vs. grade I meningioma. The top 10 genes based on average rank value exhibiting either a negative transcript LFC and positive protein LFC (high average rank value) or a positive transcript LFC and negative protein LFC (low average rank value) are highlighted in yellow and blue, respectively. (**b,c**) Top 10 genes showing discordant differential expression with negative transcript LFC and positive protein LFC (**b**) or positive transcript LFC and negative protein LFC (**c**) in grade III vs. grade I meningiomas are presented with respective LFC, rank and average rank values. Table S3B provides details of all 3598 genes common to both analyses, transcript/protein LFC, their discordant rank value and average rank. Protein LFC values were ranked in ascending order (lowest LFC assigned rank of 1), transcript LFC values ranked in descending order and average rank calculated.

Next, we explored discordant transcriptome–proteome differential expression. This approach enabled us firstly to prevent dismissal of potential targetable proteins exhibiting high protein LFC but low transcript LFC in grade III vs. grade I meningiomas, and secondly, to investigate possible inhibition of tumour suppressor translation in grade III compared to grade I displaying low protein LFC but a high transcript LFC. Again, a rank-based approach was utilised to identify genes with profound discordant expression (Figure 2a; Table S3B). The 10 genes ranked with the strongest discordant differential expression demonstrating either a subtle transcript LFC and strong positive protein LFC (high average rank value) or a positive transcript LFC and negative protein LFC (low average rank value) are shown in Figure 2b,c. Discordant genes with a high average rank included *SLC4A4*, which encodes a Na+/HCO3- co-transporter with a transcript LFC of −0.92 and protein LFC of 4.28, as well as *ACS1*, which encodes the phosphofurin acidic cluster sorting protein-1, found to have −0.51 transcript LFC and 3.34 protein LFC (Figure 2b). Displaying the lowest average rank (107.5) among discordant genes was the linker histone H1.2 gene, *HIST1H1C*, with a transcript LFC of 1.24 and protein LFC of −1.61 (Figure 2c). In addition, *NPTX1*, known to promote neurite outgrowth, showed a low average rank of 112.5 with a 1.69 transcript LFC and −1.44 protein LFC (Figure 2c). 

To explore discordant differential expression, we focused on a possible cause that could lead to observing negative transcript LFC but high protein LFC in grade III meningioma compared to grade I. Here, we hypothesised that increased protein levels may result from the plasma via the tumour vasculature and thus do not need to be correlated with increased transcript levels within the tissue. As both datasets integrated in this study captured transcripts and proteins derived not only from tumour cells but also the tumour microenvironment, we can presume that a proportion of identified proteins may have originated from the plasma. This possibility is further supported by the observation that meningiomas tend to be highly vascularised brain tumours [27]. We therefore overlapped a publicly accessible human plasma proteome reference set comprising 1929 canonical protein sequences with the top 100 discordantly expressed genes showing positive protein LFC but negative transcript LFC based on average rank (Table S3C) [28]. Following this, we identified 21 common proteins that have previously been found in human plasma, including myelin basic protein (MBP), phosphofurin acidic cluster sorting protein 1 (PACS1), amyloid-beta precursor protein (APP), desmoplakin (DSP) and lysosome-associated membrane glycoprotein 2 (LAMP2) (Table 1).

### 2.2. Functional Annotation of Grade III vs. Grade I Meningioma Differential Transcriptome–Proteome Expression

**Figure 3:**
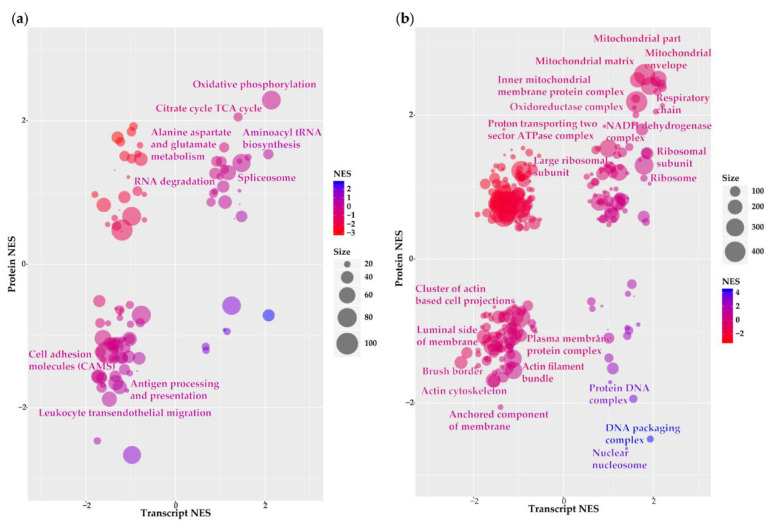
Functional annotation of grade III vs. grade I meningioma transcriptome–proteome. (**a**) Kyoto Encyclopaedia of Genes and Genomes (KEGG) pathway analysis and (**b**) gene ontology (GO) analysis of cellular component terms presenting enriched pathways/cellular components overlapping between transcript and protein datasets. Bubble plots show normalised enrichment scores (NES) of pathways/cellular components derived from Gene Set Enrichment Analysis (GSEA) of genes ranked by transcript and protein LFCs. Size and colour of each bubble represent the number of differentially expressed genes enriched in the pathway and the combined transcript and protein NES, respectively. Highlighted are pathways/cellular components with false discovery rate (FDR) < 0.2 for both transcript and protein NES. Table S4 details KEGG pathway and GO cellular component output.

Functional annotation analyses were performed to elucidate signalling pathways and biological processes associated with differentially expressed transcripts/proteins. We submitted the 3598 transcripts and proteins with corresponding LFCs to Gene Set Enrichment Analysis (GSEA) to identify overlapping enriched Kyoto Encyclopaedia of Genes and Genomes (KEGG) pathways and gene ontology (GO) terms (Table S4). Following KEGG pathway analysis, we observed 22 pathways with a positive normalised enrichment score (NES) in both datasets suggesting their enrichment in grade III compared to grade I meningioma (Figure 3a; Table S4A). Among pathways with the highest NES were numerous metabolic processes: “oxidative phosphorylation” (77 genes), “aminoacyl tRNA biosynthesis” (31 genes), “citrate cycle TCA cycle” (27 genes), “purine metabolism” (54 genes), “pyrimidine metabolism” (33 genes) and “alanine, aspartate and glutamate metabolism” (18 genes). We identified 47 pathways with a negative transcript and protein NES implying their downregulation in grade III meningioma vs. grade I (Figure 3a; Table S4A). Several pathways displaying negative NES were associated with aspects of the immune system such as “leukocyte transendothelial migration” (53 genes), “antigen processing and presentation” (31 genes), “FcγR-mediated phagocytosis” (48 genes) and “complement and coagulation cascades” (40 genes). Among the remaining pathways showing negative NES were those surrounding cellular adhesions including “adherens junction” (36 genes), “regulation of actin cytoskeleton” (105 genes), “tight junction” (54 genes) and “gap junction” (41 genes). To obtain a more comprehensive coverage of the functional composition of meningioma molecular pathogenesis, we applied a less stringent false discovery rate (FDR) of <0.2 to transcript and protein NESs across enrichment analyses. Note, an FDR threshold of <0.2 in both the transcriptomic and proteomic enrichment analyses corresponds to a combined *p*-value < 0.04 for the simultaneous observation of significant enrichment at both transcriptomic and proteomic level due to the multiplicative nature of probabilities. Subsequent to relaxation of the FDR threshold, six of the 22 upregulated KEGG pathways remained and are highlighted in Figure 3a. Again, we observed “oxidative phosphorylation” and “aminoacyl tRNA biosynthesis” suggesting that pathways related to metabolism are strongly enriched at both the transcriptome and proteome levels in grade III meningioma. Although only seven of the 48 downregulated pathways harboured an FDR < 0.2, these included pathways related to the previously identified biological themes of cellular adhesion with “cell adhesion molecules (CAMs)” (50 genes) and of the immune system with “leukocyte transendothelial migration” and “antigen processing and presentation” pathways all significantly reduced in grade III vs. grade I meningiomas (Figure 3a). This observation confirms recent findings based on RNA-seq data and tissue staining by Viaene and colleagues that immune activity is increased in grade I compared to grade II or III meningioma [13].

In addition to annotation with enriched KEGG pathways, we performed GO cellular component analysis to characterise the localisation of differentially expressed gene products within the cellular anatomy. Among 84 terms exhibiting concordant positive NESs, 19 harboured significant enrichment (FDR < 0.2) with over half of these related to mitochondria including “mitochondrial envelope”, “mitochondrial matrix”, “respiratory chain” and “NADH dehydrogenase complex” (Figure 3b; Table S4B). These findings were analogous to our previous pathway observations comprising many enriched metabolic processes (Figure 2a). Remaining cellular components found to be significantly enriched among upregulated transcripts/proteins of grade III meningioma were largely associated with ribosomes. GO analysis revealed 85 terms demonstrating concordant negative NESs of both downregulated transcripts and proteins (Table S4B). Eight terms were classed as significant (FDR < 0.2) of which at least two indicated a decline in cytoskeletal organisation such as “actin cytoskeleton” (200 genes) and “actin filament bundle” (31 genes) (Figure 3b).

### 2.3. Validation of Concordant and Discordant Expression in Grade III Meningioma

**Figure 4:**
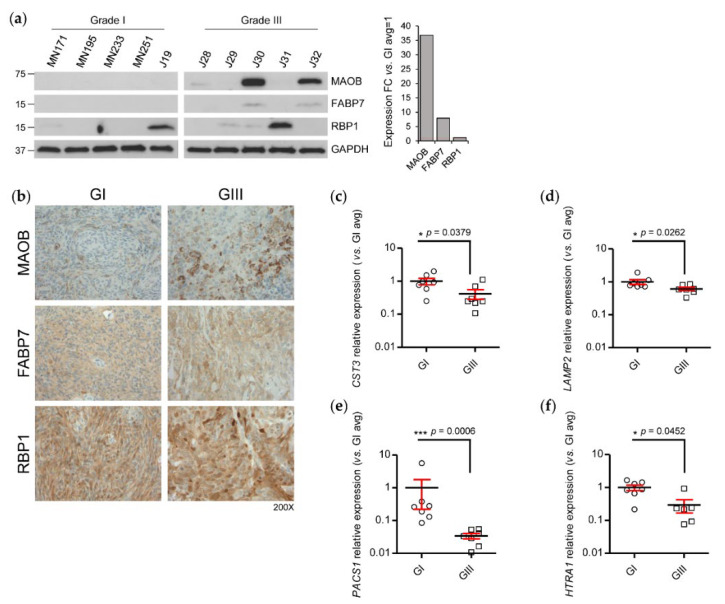
Validation of concordantly and discordantly expressed differentially expressed proteins and transcripts in grade III vs. grade I meningiomas. (**a**) Western blot (WB) analysis of the proteins MAOB, FABP7 and RBP1 that demonstrated a concordant increase in expression following transcriptome–proteome integration in meningioma tissue lysates. WB quantification of average densities of MAOB, FABP7 and RBP1 in grade III meningioma compared to average densities in grade I is shown. GAPDH was used as loading control. Grade I *n* = 5, grade III *n* = 5. (**b**) Immunohistochemistry validation of MAOB, FABP7 and RBP1. Representative images show immunohistochemical staining in grade I and grade III meningioma. Table S5 details the full list of specimens and corresponding semi-quantitative assessment. Grade I *n* = 10, grade III *n* = 10. RT-qPCR validation of discordantly expressed transcripts (**c**) *CST3* (**d**) *LAMP2* (**e**) *PACS1* and (**f**) *HTRA1* that exhibited negative transcript but positive protein LFC in grade III meningioma compared to grade I following transcriptome–proteome integration. Grade I *n* = 7, grade III *n* = 7. Statistical significance was determined by Mann–Whitney test * *p* ≤ 0.05; *** *p* ≤ 0.001.

Subsequent to our integrated transcriptome–proteome analysis and functional annotation of grade III vs. grade I meningioma, we chose to validate a subset of concordant and discordant transcript/protein expression using, where available, an independent sample cohort of meningioma tissue. In addition, we endeavoured to select candidates for experimental validation that to date had not yet been explored in meningiomas or where previously studied, demonstrated a conflicting observation to our data presented here and thus we investigated further using an alternative technique. First, we validated the concordant differential expression of monoamine oxidase type B (MAOB) (transcript LFC 0.7, protein LFC 3.19), fatty acid-binding protein 7 (FABP7) (transcript LFC 1.63, protein LFC 6.68) and retinol-binding protein 1 (RBP1) (transcript LFC 1.12, protein LFC 3.44) at the protein level by Western blot (WB) using an independent cohort of five meningioma tissues of both grade I and grade III. We observed an average overexpression of ~37 folds for MAOB and ~eight folds for FABP7 in grade III tumours compared to grade I, whilst RBP1 average grade III expression was analogous to that of grade I (Figure 4a). WB validation displayed varying immunoreactivity across samples, indicating some underlying heterogeneity of meningioma (Figure 4a). In addition to WB, we performed experimental validation of these concordantly expressed proteins by immunohistochemistry (IHC). Importantly, this technique allowed us to expand the sample cohorts to comprise of 10 sections per grade. MAOB showed a distinct increase in expression in grade III meningiomas consistent with WB findings, generally displaying moderate immunostaining, whereas grade I tumours showed either weak or absent expression (Figure 4b; Table S5). Here, it is important to note that as our validation studies were performed on tumour tissue, any expression observed for our candidate proteins may not solely have originated from meningioma cells but also from cells of the tumour stroma. As an example, we stained the grade III meningioma, J10, for the common macrophage marker, CD68 (Figure S2). Although we detected some CD68^+^ cells, the widespread expression of MAOB throughout the tissue suggests that the majority of immunostaining is genuinely derived from meningioma cells themselves. FABP7 and RBP1 immunostaining were detected but variable within each grade and demonstrated no clear difference between the grades (Figure 4b; Table S5). Next, we performed RT-qPCR in seven grade III vs. seven grade I meningioma tissues to validate discordant differential expression at the transcript level of selected genes that exhibited negative transcript but high protein LFC and were identified in a human plasma proteome reference set. Following RT-qPCR, we identified a significant reduction in relative expression for *CST3*, *LAMP2*, *PACS1* and *HTRA1* in grade III meningioma compared with grade I compatible with our hypothesis that increased abundance of these proteins in the tumour originates from the blood circulation (Figure 4c–f).

### 2.4. Drug–Gene Interaction Analysis

**Figure 5:**
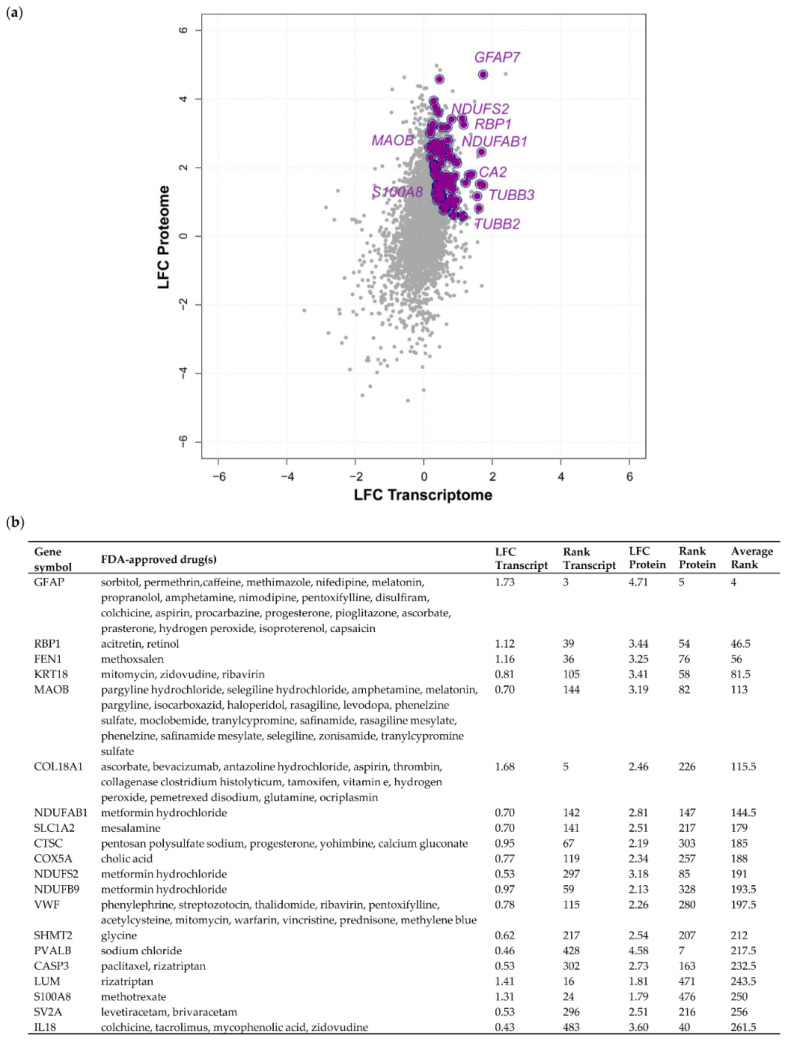
The druggable grade III meningioma proteome. (**a**) Proteins with increased concordant expression in grade III vs. grade I meningiomas also associated with Food and Drug Administration (FDA)-approved drugs are displayed. (**b**) Top 20 concordantly expressed proteins based on average rank value and their associated FDA-approved drugs following submission to the drug–gene interaction database (DGIdb, v3.0). Table S6 provides details of transcripts/proteins associated with FDA-approved drugs.

In order to identify Food and Drug Administration (FDA)-approved drugs predicted to interact with any of the 3598 proteins of our transcriptome–proteome analysis, we utilised the drug prediction database, DGIdb [29]. Although many pure transcriptomic analyses have previously harvested genes with promise as therapeutic targets of meningioma, confirmation of simultaneous dysregulation of protein expression is frequently missing. As protein abundance correlates but does not fully mirror transcript abundance, the value of drug target prediction based on transcriptomic data therefore warrants caution. We reasoned that by focusing on transcripts/proteins with concordant upregulation in grade III meningioma across both datasets, we were able to identify candidates with the strongest potential as drug targets. Following submission of the 3598 proteins to the DGIdb, 685 proteins, approximately 20%, were found to interact with one or more FDA-approved drugs and, of these, 264 displayed a concordant increase in transcript and protein expression in grade III vs. grade I meningiomas (Table S6). In addition to the previously validated proteins MAOB and RBP1, proteins displaying strong concordantly increased expression associated with FDA-approved drugs included the calcium and zinc-binding protein S100-A8 (S100A8), microtubule proteins tubulin beta-3 chain (TUBB3) and tubulin beta-2B chain (TUBB2B), carbonic anhydrase 2 (CA2), involved in maintaining pH homeostasis and multiple subunits of respiratory complex I such as NDUFAB1, NDUFS2 and NDUFB9 (Figure 5; Table S6).

## 3. Discussion

Meningioma are the most frequent primary intracranial tumour. Treatment of high-grade meningioma has remained a challenge due to their aggressive course, tendency to recur and low overall survival rates [4]. Drugs holding therapeutic efficacy in the treatment of these aggressive tumours are still lacking [2]. Here, we combined and analysed the transcriptome–proteome data of grade III meningioma compared to grade I to unravel the diverging molecular landscapes of these tumour phenotypes and identify potential grade III therapeutic targets and biomarkers. Although previous studies have provided much insight into the omics characterisation of these tumours [10,22,25], a comparison of differential expression at the transcript level and protein level, to our knowledge, has yet to be performed.

We first established transcripts and proteins common to transcriptome and proteome analyses that were previously published but extended for this study. Next, we integrated transcript and protein LFC between grade I and grade III meningioma tissues using a rank-based approach. By observing expression across these two complementary molecular levels, we were able to distinguish between genes displaying concordant changes in transcript and protein abundance from those with discordant changes. Such distinction can guide at better understanding the dysregulation of genes. Following integration, we found 3598 transcripts/proteins to be common among both omics platforms with LFCs displaying a moderate, although highly significant Pearson’s correlation coefficient (*r* = 0.35; *p* = 3.6 × 10^−104^). Previous in vitro mRNA/protein abundance studies using mammalian cancer cell lines, including glioblastoma, epidermoid carcinoma and osteosarcoma, have displayed correlation coefficients of between 0.58 and 0.63, as well as 0.54, in medulloblastoma when measured in the same samples [30,31]. The lower correlation coefficient in our study compared to these in vitro studies was expected, as our analysis was based on tumour tissue samples in contrast to the relatively homogenous cellular populations observed in cell lines. Further, our cohorts were independent but comparable in respect to grade composition, and samples from recurrent cases were excluded.

Among the top genes with concordant upregulated expression at the transcript and proteomic level in grade III vs. grade I meningiomas was *AGR2*, a metastatic oncogene coding for the disulfide isomerase endoplasmic reticulum protein, anterior gradient 2 homolog (AGR2). Overexpression of AGR2 has been linked to the proliferation, migration and invasion of several cancers [32,33]. Indeed, AGR2 has previously been implicated as a novel stem cell biomarker overexpressed in a subset of aggressive primary meningioma cell lines [34]. Hence, combined, these data suggest AGR2 may be a contributor to an aggressive meningioma phenotype and should be considered as a possible molecular marker or therapeutic target on the transcript or protein level. Similarly, the fatty acid-binding protein FABP7 was also among the top concordantly expressed genes with its overexpression previously reported to enhance the proliferation and migration of many tumour types [35]. Our validation studies showed FABP7 expression increased by WB but not by IHC in grade III meningioma compared to grade I. As the lack of detection by the latter method might be explained by the known limits of IHC quantification, we would not discard FABP7 on this basis. Moreover, two previous studies have reported a significant correlation between increasing FABP7 expression and increasing meningioma grade following IHC [36,37]. Taken together, these findings provide a strong rationale to investigate the potential role of FABP7 in meningioma pathogenesis. Interestingly, FABP7 acts as an intracellular transporter of the antinociceptive endocannabinoid, anandamide, to the fatty acid amide hydrolase for its inactivation, which has led to the development of readily available FABP7 inhibitors for their analgesic and anti-inflammatory properties [38,39]. In tumourigenesis, the molecular mechanisms surrounding the involvement of FABP7 remain unclear. Yet, following FABP7 depletion in several cancer cell lines, proliferation and migration are reduced [40,41,42,43], inferring that exploring FABP7 inhibitors may hold promise as a future targeted therapy of meningioma. 

We also observed concordantly downregulated transcripts/proteins of grade III meningioma compared to grade I, providing valuable biomarker candidates and insights into tumorigenesis. Genes found to be downregulated in grade III meningioma with reported tumour suppressor roles in various other cancers included *FBLN1*. As an extracellular matrix (ECM) protein of the fibulin family, FBLN1 functions in the organisation of ECM supramolecular structures, thereby playing a role in the regulation of cell morphology, growth, adhesion and motility [44]. Downregulation of FBLN1 expression has been shown to be associated with tumour progression and act as a prognostic factor in many cancers such as gastric, renal cell carcinoma, hepatocellular carcinoma and lung adenocarcinoma [45,46,47,48]. In meningioma, overexpression of FBLN1 has previously been identified in the fibroblastic grade I histological subtype, characterised by a rich collagen matrix, when compared to the grade I meningothelial subtype [11,49]. Moreover, Fevre et al. (2009) suggested FBNL1 acts as a tumour suppressor in fibroblastic meningioma whilst alternative suppressors are at play in the meningothelial subtype [11]. Hence, reduced FBLN1 expression in grade III vs. grade I meningiomas detected in this study supports the notion that loss of this protein may contribute to a malignant phenotype of these tumours. Interestingly, in many studies, downregulation of FBLN1 results from hypermethylation of its promoter [46,47], indicating the importance of expanding upon the omics platforms integrated here to those such as the epigenome, in order to dissect the molecular mechanisms underlying the observed dysregulation. In addition to FBLN1, we identified concordant downregulation of LEPR that binds the multifunctional hormone ligand, leptin, primarily synthesised by adipose tissue [50]. This receptor–ligand complex predominantly acts to regulate adipose tissue mass and energy homeostasis through various kinase signalling cascades [50]. Although found to be overexpressed in some cancers [51,52], LEPR expression was downregulated in high grade meningioma in this study as well as in two previous meningioma studies [12,26]. Mechanisms underlying LEPR downregulation and its association with increased tumour progression are not yet understood. However, as a prognostic biomarker of disease aggressiveness, LEPR has also shown promise in several other cancers including prostate, endometrial and thyroid, indicating that its value in meningioma prognosis ought to be investigated further [53,54,55].

Subsequent to exploring concordantly up and downregulated transcripts/proteins, we focused on those showing discordant expression in grade III vs. grade I meningiomas. As discussed earlier, changes in transcript and protein abundance generally show only limited correlation. Various biological and technical parameters influence mRNA–protein correlation such as mRNA degradation rate, ribosomal density, protein half-life, compartmental transcript sequestration, secondary RNA structure, measurement error and noise [56,57]. Thus, we elected not to exclude discordant expression as these trends may also harbour therapeutic targets and reveal further insight into altered gene expression between low- and high-grade meningioma. Among those transcripts/proteins displaying the strongest discordant expression with high protein but low transcript LFC in grade III tumours were the Na^+^/HCO_3_^-^ co-transporter SLC4A4, previously shown to contribute to the proliferation, migration and invasion of a breast cancer cell line [58], and the phosphofurin acidic cluster sorting protein-1, PACS1, recently revealed to function in the nucleus as an epigenetic regulator to promote oncogenic replication and progression [58,59]. To further investigate this discordant trend, we hypothesised that tumour vasculature may play a role in delivering proteins that are not upregulated on the transcript level in the tissue itself, to the tumour tissue. Intersection of discordant genes with a publicly accessible human plasma proteome reference set led to the identification of 21 common proteins [28]. We selected four of these 21 proteins: PACS1, LAMP2, high-temperature requirement A serine peptidase 1 (HTRA1) and cystatin C (CST3), and validated a significant reduction in transcript LFC for each in grade III vs. grade I meningiomas by RT-qPCR. HTRA1 is a ubiquitously expressed serine protease known to be secreted but also found to have an intracellular presence [60]. The role of HTRA1 in cancer has been defined as both tumour suppressive and pro-oncogenic [60]. In meningioma, Önder et al. (2015) used IHC to report a correlation of decreasing cytoplasmic HTRA1 expression with increasing grade and recurrence, proposing its use as a behavioural marker in these tumours [61]. Conversely, our analyses revealed an opposing trend of increasing protein expression in high-grade meningioma, yet we also observed downregulation of HTRA1 transcript LFC. Interestingly, a previous study identified HTRA1 expression in the tumour stroma to regulate Notch signalling, thereby coordinating angiogenesis and enhancing tumour progression [62]. Therefore, in line with our hypothesis, HTRA1 may also derive from the tumour microenvironment of fast-growing grade III meningioma to promote angiogenesis. Unlike HTRA1, LAMP2 has remained unreported in meningioma until now. LAMP2 is a major glycoprotein component of the lysosomal membrane that functions in autophagy and maintaining lysosomal integrity [63]. Former studies have linked elevated protein expression of LAMP2 to increased metastatic potential and poor prognosis in malignancies of the colon, prostate and esophagus [64,65,66]. Here, we again hypothesised increased LAMP2 protein abundance in grade III meningioma may arise from the plasma, a theory partially supported by Gabriele et al. (2018), who detected a significant increase in LAMP2 protein in prostate cancer patients’ sera compared to control using glycopeptide enrichment and targeted LC-MS/MS [67]. Thus, subject to further investigation, discordantly expressed transcripts/proteins such as HTRA1 and LAMP2 could hold promise as diagnostic blood biomarkers for high-grade meningioma.

In addition to identifying individual differentially expressed transcripts/proteins in high-grade meningioma that may be of clinical relevance, we also explored their associated signalling pathways and cellular localisations. Predominant biological themes arising from concordantly upregulated grade III transcripts/proteins included metabolic processes, such as the TCA cycle and oxidative phosphorylation, which were echoed by an enrichment in mitochondrial localisation. To date, few studies have ventured to define the metabolic profile of meningioma. Yet, Vasudevan et al. (2018) identified the transcriptome of male patients with high-grade meningioma also to be enriched in metabolic and oxidative phosphorylation genes following KEGG pathway analysis [25]. Collectively, these findings warrant investigation into the presence of altered metabolic pathways in meningioma as well as grade-specific metabolic reprogramming that may be exploited for targeted therapy. Concordantly upregulated transcripts/proteins were also enriched in pathways and cellular locations related to RNA metabolism. Interestingly, we previously observed an association with RNA metabolism, including ribosomes and splicing, by both global and phosphoproteomic grade III-specific analyses [19]. Furthermore, several individual transcripts/proteins/phosphoproteins reoccurred throughout these grade III-specific analyses, such as members of the DEAD-box family of RNA helicases, including DDX5, DDX6 and DDX42, along with components of the spliceosome including SNRPE and SNRPF. Together, these commonalities reinforce the importance of studying the role of RNA metabolism in meningioma aggressiveness and may also lead to the discovery of grade-specific splice variants with potential as biomarkers or therapeutic targets. Concordantly downregulated grade III transcripts/proteins were enriched in pathways and cellular locations related to cellular adhesion and the immune system. Interestingly, among genes enriched in the “cell adhesion molecules (CAMs)” pathway was *cell adhesion molecule 1* (*CADM1*), whose loss of protein expression has formerly been found to correlate with increasing meningioma grade and reduced patient survival [68]. CADM1 is a transmembrane glycoprotein with several tumour suppressor roles including modulation of cell cycle progression, induction of apoptosis, activation of immunosurveillance responses as well as enhancement of cell–cell adhesion through its extracellular domain [69]. Loss of CADM1 expression by promoter hypermethylation or loss of heterozygosity (LOH) has been strongly linked to poor prognosis, tumour progression, invasion and metastasis in many cancers, and has thus become an attractive diagnostic and prognostic biomarker [69]. Further examination into downregulated cell adhesion pathways and their components may therefore lead to novel biomarker discoveries, and if integrated with methylomic profiling could promote the therapeutic use of DNA methyltransferase inhibitors in high-grade meningiomas. 

Finally, we explored abundant grade III proteins already associated with one or more FDA-approved drugs that could hold therapeutic efficacy against these tumours. Among the 264 concordantly upregulated genes found to be associated with at least one drug were MAOB and RBP1, upon which we performed validation studies. Here, it is important to note that in the absence of a combined ranking of LFCs, genes such as MAOB and RBP1 would not have shown enough prominent differential expression in either dataset alone to warrant their validation, demonstrating the importance of integrated analyses. While RBP1 did not exhibit a rise in protein expression between grade III and grade I meningiomas, MAOB presence was found to be higher in grade III tumours by both WB and IHC. MAOB is a member of the monoamine oxidases family that catalyse the oxidative deamination of several monoamine neurotransmitters. In glioblastoma, high expression and activity of MAOB has led to the exploration of an MAOB-activated pro-drug and selective MAOB inhibitors as potential treatments [70,71]. Although a pathophysiological role of MAOB in high-grade meningiomas has yet to be determined, its association to 19 FDA-approved drugs suggests MAOB may prove to be a strong candidate as a molecular target in these tumours.

For the future we anticipate that our integrative approach will be considerably boosted by the availability of multi-omics data from the same patient cohort. Our current study was conducted on the basis of data integration over two different cohorts, which introduced additional variability and limited the fidelity in detection of concordant or discordant changes. Removing the inter-cohort variability will enable a more accurate dissection of molecular changes of heterogeneous tumour types such as meningioma grade II tumours. It will also facilitate the identification of prognostic markers that can stratify samples within the same grade, as our previous study and a recent study by Viaene and colleagues implied [12,13].

In summary, we performed an integrated multi-omics study comprising transcriptomic and proteomic data from two independent analyses of meningioma tissue. We identified previously described, as well as novel, transcripts/proteins with either concordant up- or downregulated differential expression between grade III and grade I meningiomas. Moreover, we acknowledged discordant differential expression and suggested an attractive hypothesis explaining the observation of increased protein abundance despite lower transcript levels for a sub-group of genes. The corresponding transcript levels were validated for *PACS1*, *LAMP2*, *HTRA1* and *CST3* in meningioma tissue. Prospective proteomic studies of meningioma patient plasma will be required to further strengthen the validity of these potential diagnostic blood markers. Our results also underlined biological themes such as metabolic reprogramming, RNA metabolism, immune regulation and cellular adhesion that, with in-depth evaluation, may elucidate targetable pathways in high-grade meningiomas. Lastly, we revealed a substantial amount of concordantly upregulated proteins to be associated with one or more FDA-approved drugs, among which was the validated protein, MAOB, encouraging drug repurposing for meningioma treatment. Overall, we demonstrate the added value gained by combining independent omics datasets to enhance our current view of the molecular landscape of meningioma and to highlight candidates, which upon further investigation, may harbour potential as biomarkers or therapeutic targets of grade III meningioma.

## 4. Materials and Methods 

### 4.1. Data Acquisition and Processing

Transcriptome and proteome data of meningioma tissue from two independent studies were integrated in the current analysis. The transcriptomic dataset produced by Schmidt et al. (2016) was accessed via the NCBI Gene Expression Omnibus under the GEO accession number GSE74385 and covered a microarray analysis of 62 meningioma tissues encompassing all WHO grades obtained using the Human HT-12 v4 BeadChip array (illumina^®^) [12]. Ten additional meningioma tissues were obtained under the GEO accession number GSE149923 and combined dataset vsn normalised using the vsn package (v3.50.0) in the R (v3.5.3) programming environment. Meningiomas were further subgrouped based on their tendency to recur: NR = no recurrence observed within a period of at least 36 months; R = recurrence of the same WHO grade following complete resection; M = malignant progression of tumour with recurrence at a higher WHO grade following complete resection; NA = no further follow-up available or incomplete resection. For the present study, we compared the transcriptome of primary meningioma grade I NR (*n* = 13) (referred to as grade I for the purposes of this study) with that of all primary meningioma grade III (*n* = 20). LFC and adjusted *p*-values were retrieved between both groups by model fit and contrasts using the limma R package (v3.34.8). The global proteomics dataset produced by Dunn et al. (2018) (PRIDE repository: PXD007073) using LC–MS/MS was processed as previously described [19]. Label-free quantification (LFQ) values were log transformed and LFC between grade III (*n* = 6) and grade I meningioma (*n* = 8) calculated in R (v3.5.1). Tumours were classified by grade according to the 2007 WHO Classification of Tumours of the Central Nervous System. Clinical and histological details of the meningioma specimens analysed in the transcriptomic and proteomic studies are presented in Table S1.

Density plot displaying the distribution of transcriptomic and proteomic LFC of grade III vs. grade I meningiomas was generated in R covering LFC values for 31,385 transcripts and 3846 proteins. UniProt IDs were mapped to corresponding Entrez Gene IDs using the annotation provided in the Bioconductor org.Hs.eg.db package (v3.10). UniProt IDs that could not directly be mapped were alternatively converted to HUGO Gene Nomenclature Committee (HGNC) IDs and then to Entrez Gene IDs by the HGNC database. Subsequently, Entrez Gene IDs common to both datasets were identified. Due to substantial differences observed from the underlying distribution of transcriptomic or proteomic LFC values, a rank-based approach was used to compare transcriptomic and proteomic changes [72]. To determine transcripts/proteins with concordant expression, we first ranked transcript and protein LFC values separately in descending order (highest LFC assigned rank of 1) followed by calculating the average rank. Concordant upregulation was indicated by a low average rank while a high average rank pointed to concordant downregulation. Discordant protein expression was identified as follows: protein LFC values were ranked in ascending order (lowest LFC assigned rank of 1), whereas transcript LFC values ranked in descending order (as for concordant expression). After calculating the average rank, we observed low average ranks for genes whose corresponding proteins were downregulated while their transcripts were upregulated. In contrast, large average ranks were representative of genes with upregulated proteins and downregulated transcripts.

A transcriptome vs. proteome LFC scatterplot was created and Pearson’s correlation coefficient determined using the basic R functions. Discordantly expressed proteins were intersected with the published Human Plasma Peptide Atlas consisting of 1929 canonical protein sequences to identify those also present in human plasma [28]. To identify genes targeted by FDA-approved drugs, gene symbols were submitted to the drug–gene interaction database (DGIdb) (v3.0, www.dgidb.org) and the “Food and Drug Administration (FDA) approved” filter was applied [29].

### 4.2. Functional Enrichment Analyses

Enrichment of biological processes and pathways was investigated based on gene annotation obtained using Gene Set Enrichment Analysis (GSEA) (v4.0.3) of the Kyoto Encyclopaedia of Genes and Genomes (KEGG) and Gene Ontology (GO) databases. To assess whether gene sets defining biological processes or pathways showed a tendency for up or downregulation at the transcriptomic or proteomic level, GSEA was applied to pre-ranked lists of genes or proteins [73]. Statistical significance was estimated using a background set of 1000 random permutations. KEGG pathways and GO terms of transcripts and proteins were considered significantly enriched with false discovery rate (FDR) < 0.2 and visualised as bubble plots using the ggplot2 R package (v3.2.1). For KEGG pathway enrichment, disease-specific pathways were excluded from the analysis to facilitate focus on enriched signalling and metabolic pathways with more biological relevance to meningioma pathology.

### 4.3. Clinical Material

Anonymised meningioma samples under the “J” series were provided by the BRain Archive and Information Network (BRAIN UK) under ethical approval by the South West research ethics committee (REC No: 14/SC/0098; IRAS project ID: 143874, BRAIN UK Ref: 15/011). Anonymised “MN” meningioma samples were collected under ethical approval by the South West research ethics committee (REC No: 14/SW/0119; IRAS project ID: 153351) and local research and development approval (Plymouth Hospitals NHS Trust: R&D No: 14/P/056 and North Bristol NHS Trust: R&D No: 3458). Clinical and histological details of the meningioma specimens analysed in the validation studies are presented in Table S1. 

### 4.4. Cell Culture and Western Blotting

Human meningeal cells (HMC) were obtained from ScienCell^™^ and cultured in the manufacturer’s recommended medium and growth supplements at 37 °C in humidified 5% CO_2_. Cells and meningioma tissue were lysed as previously described [19] and protein concentration determined using the Pierce^™^ BCA Protein Assay Kit (Thermo Fisher Scientific, Oxford, UK). Western blotting was performed by separating proteins on 8% or 15% Laemmli SDS-PAGE, depending upon the molecular weight of the protein of interest and proteins transferred to a polyvinylidene difluoride membrane (Immun-Blot^®^ PVDF membrane, Bio-Rad, London, UK). Membranes were blocked for 1 hr at room temperature with 5% skimmed milk in phosphate buffered saline (PBS) with 0.05% Tween-20, before incubation with specific primary antibodies overnight (O/N) at 4 °C. Primary antibody details are listed in Table S2. Specific antigen–antibody interaction was detected with anti-mouse and anti-rabbit secondary antibodies conjugated to horseradish peroxidise (Bio-Rad). Detection was achieved using the ECL or ECL Plus Western Blotting substrate (Pierce). Membranes were exposed to Amersham Hyperfilm ECL (GE Healthcare Life Sciences, Chalfont St Giles, UK). Films were scanned at a resolution of 600 dpi using a HP Scanjet 2400. Immunoreactive bands were quantified using the ImageJ processing program and each band normalised to corresponding GAPDH loading control values. The average value for grade I samples was then calculated and taken as the reference value of one for quantification. All samples were each divided by the average grade I value. Average quantification values for grade III meningiomas were then plotted in a bar chart representing expression fold change (FC) vs. grade I = 1.

### 4.5. Immunohistochemistry

Four-micrometre formalin-fixed paraffin-embedded sections were de-waxed, rehydrated and incubated with primary antibodies at room temperature O/N using antigen retrieval methods as previously described [74]. Primary antibody details are listed in Table S2. Proteins were visualised with the Vectastain Universal Elite ABC kit (Vector Laboratories Ltd., Burlingame, CA, USA). Slides were counterstained with haematoxylin (Sigma-Aldrich, Gillingham, UK). As a control, sections were incubated with omission of the primary antibody. Immunohistochemical results were reviewed by consultant neuropathologist, Hilton, D.A. Semi-quantitative assessment of staining intensity was scored as follows: 0 if negative; 1 if low immunoreactivity; 2 if moderate; 3 if high.

### 4.6. RNA Isolation and Quantitative PCR

Total RNA was extracted from HMC and meningioma tissue using the QIAzol^®^ Lysis Reagent (Qiagen, Manchester, UK) according to the manufacturer’s instructions. RNA quality and concentration were assessed using the NanoDrop^™^ 2000 (Thermo Fisher Scientific, Oxford, UK). One µg of total RNA was reverse transcribed using a High-Capacity cDNA Reverse Transcription Kit (Applied Biosystems^™^, Thermo Fisher Scientific, Oxford, UK). Quantitative PCR (qPCR) was performed on a Real-Time qPCR LightCycler^®^ 480 System (Roche, Welwyn Garden City, UK) using TaqMan^™^ Fast Advanced Master Mix and the corresponding TaqMan^®^ Gene Expression Assay (Assay IDs: CST3 Hs00969174_m1; PACS1 Hs00216026_m1; LAMP2 Hs00174474_m1; HTRA1 Hs01016151_m1; GAPDH Hs02786624_g1) (Applied Biosystems^™^, Thermo Fisher Scientific, Oxford, UK). Thermal cycling conditions were as follows: 2 min. at 95 °C, 45 cycles of amplification with denaturation at 95 °C for 15 sec and annealing and extension at 60 °C for 1 min, and cooling at 40 °C for 30 s. Relative gene expression was analysed in triplicate using the 2^™ΔΔC^_T_ method normalised to GAPDH and relative to HMC [75]. A Mann–Whitney test was performed using GraphPad Prism version 5.00 for Windows to determine the significance of changes in relative expression between grade I and grade III meningiomas for each gene.

## 5. Conclusions

Here, we present for the first time the integrated transcriptome–proteome landscape of aggressive grade III meningiomas compared to benign grade I meningiomas. Dataset integration led to the identification of 3598 shared transcripts and proteins. Concordant and discordant differential expression analyses revealed proteins including MAOB, PACS1, LAMP2, HTRA1 and CST3 to show potential as grade III-specific biomarkers or therapeutic targets. In addition, we detected consistent enrichment in pathways related to energy and RNA metabolism across transcript and protein levels, whilst concordantly downregulated transcripts/proteins were associated with immune regulation and cellular adhesion. Further studies are warranted to determine the role and therapeutic plausibility of these proteins and pathways in grade III meningiomas. 

## Figures and Tables

**Table 1:** Twenty-one of the top 100 discordantly expressed genes (positive protein LFC but negative transcript LFC) also found to be present in the published Human Plasma Peptide Atlas consisting of 1929 canonical protein sequences [28]. Shown are overlapping genes with respective LFC, rank and average rank values. Table S3C details discordantly expressed proteins also identified in the human plasma proteome reference set.

Gene Symbol	Protein Name	LFC Transcript	Rank Transcript	LFC Protein	Rank Protein	Average Rank
MBP	Myelin basic protein	−1.21	3521	2.05	3234	3377.5
PACS1	Phosphofurin acidic cluster sorting protein 1	−0.51	3218	3.34	3534	3376
APP	Amyloid-beta precursor protein	−0.77	3395	2.26	3318	3356.5
DSP	Desmoplakin	−1.43	3544	1.48	2938	3241
FCGBP	IgGFc-binding protein	−0.46	3156	2.11	3264	3210
LAMP2	Lysosome-associated membrane glycoprotein 2	−0.71	3369	1.64	3043	3206
BOLA2B	BolA-like protein 2	−0.28	2823	3.30	3532	3177.5
PTPRF	Receptor-type tyrosine-protein phosphatase F	−1.03	3487	1.33	2828	3157.5
DCN	Decorin	−0.77	3400	1.39	2875	3137.5
AK3	GTP:AMP phosphotransferase AK3, mitochondrial	−0.51	3219	1.58	3006	3112.5
HTRA1	Serine protease HTRA1	−1.47	3550	1.12	2670	3110
OGN	Mimecan	−0.89	3445	1.19	2731	3088
GOLIM4	Golgi integral membrane protein 4	−0.73	3379	1.25	2771	3075
LAMB2	Laminin subunit beta-2	−0.64	3331	1.29	2799	3065
ITM2B	Integral membrane protein 2B	−0.63	3319	1.24	2766	3042.5
SDHB	Succinate dehydrogenase [ubiquinone] iron-sulfur subunit, mitochondrial	−0.31	2870	1.90	3175	3022.5
CST3	Cystatin-C	−0.38	3034	1.51	2962	2998
LTBP4	Latent-transforming growth factor beta-binding protein 4	−0.53	3239	1.21	2745	2992
PPP3CA	Serine/threonine-protein phosphatase 2B catalytic subunit alpha isoform	−0.54	3245	1.15	2694	2969.5
HEXA	Beta-hexosaminidase subunit alpha	−0.27	2765	1.87	3163	2964
PCSK1N	ProSAAS	−0.83	3426	0.91	2483	2954.5

## Supplementary Materials

The following are available online at https://www.mdpi.com/2072-6694/12/11/3270/s1, Figure S1: Distribution of GIII vs. GI meningioma log_2_ fold changes (LFC) derived from transcriptomic and proteomic datasets, Figure S2: Expression of MAOB and CD68 in the grade III meningioma sample J10, Figure S3: Uncropped Western blot gels for Figure 4A, Table S1: Clinical and histological data of the meningioma specimens analysed in either the microarray or LC-MS/MS discovery sets and those analysed in the independent validation studies, Table S2: Primary antibody specifications used in Western blotting and immunohistochemistry, Table S3: (A): Transcript and protein ranking based on grade III vs. grade I meningioma LFC, (B): Discordant transcriptome–proteome expression based on GIII vs. GI meningioma LFC, (C): Discordantly expressed proteins identified in plasma, Table S4: (A): Overlapping KEGG pathway enrichment analysis of transcripts and proteins, (B): Overlapping Hallmark gene sets enrichment analysis of transcripts and proteins, Table S5: Semi-quantitative scoring of immunohistochemical staining for validation of concordantly expressed proteins, Table S6: (A): Genes predicted to interact with FDA-approved drugs (grade III vs. grade I meningioma LFC), (B): Discordantly expressed proteins identified in plasma and their predicted interaction with FDA-approved drugs, (C): Details of FDA-approved drugs predicted to interact with genes of the transcriptome–proteome GIII vs. GI meningioma dataset.
