# Supplementary material for: Integration and Comparison of Transcriptomic and Proteomic Data for Meningioma
Source: Cancers (Basel). 2020 Nov 5;12(11):3270. doi: 10.3390/cancers12113270 (PMC7694371; doi:10.3390/cancers12113270)
Supplement: Supplementary file 1 [file cancers-12-03270-s001.zip › cancers-99702-proofreading suppl/cancers-977702-proofreading suppl.docx]

Supplementary Materials

Integration and Comparison of Transcriptomic and Proteomic Data for Meningioma

Jemma Dunn, Vasileios P. Lenis, David A. Hilton, Rolf Warta, Christel Herold-Mende, C. Oliver Hanemann and Matthias E. Futschik


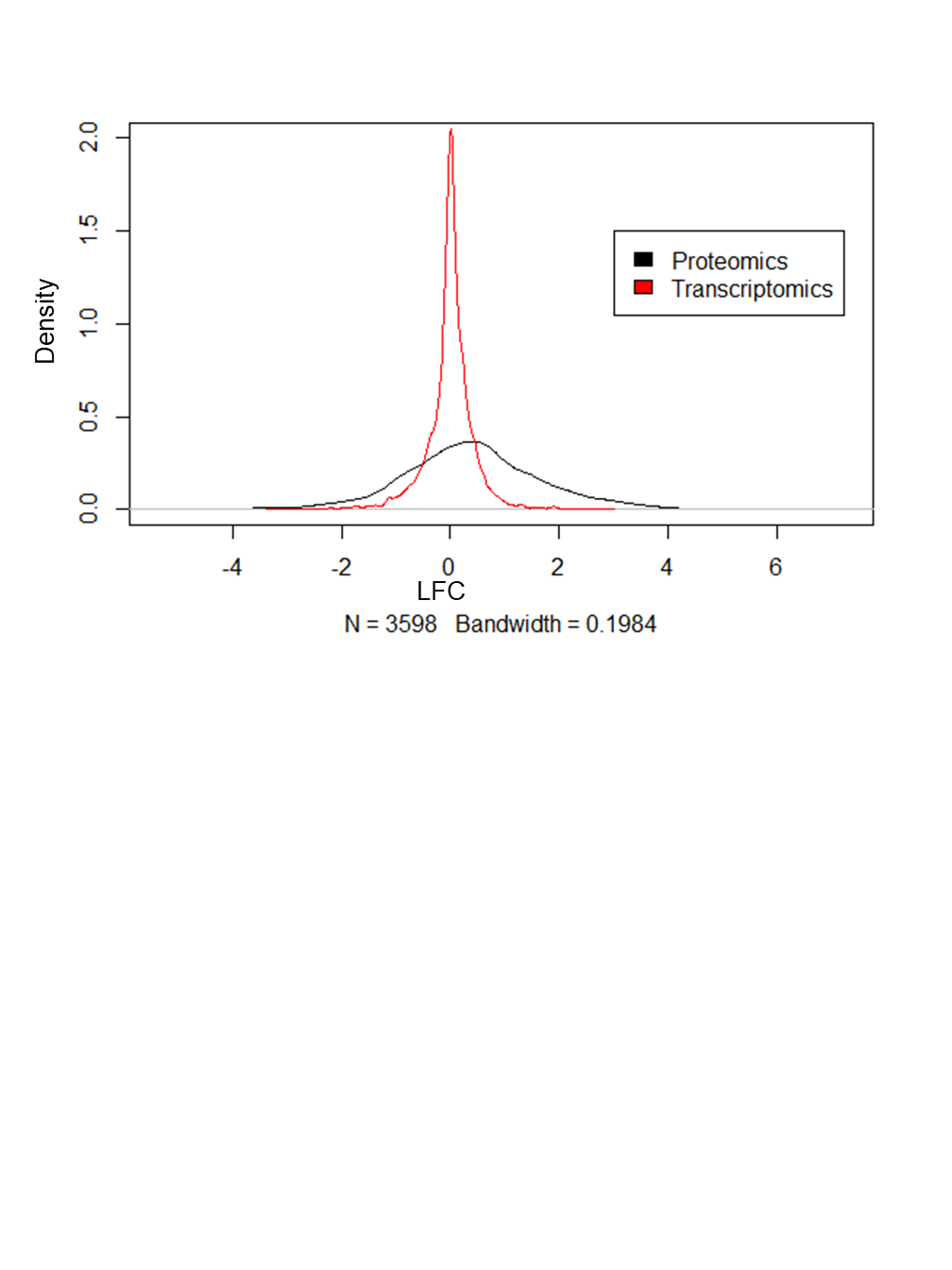


**Figure S1.** Distribution of GIII vs. GI meningioma log_2_ fold changes (LFC) derived from transcriptomic and proteomic datasets. Density plot displaying LFC distribution of GIII vs. GI meningioma transcriptome analysis (mean 0.00) and of GIII vs. GI proteome analysis (mean 0.41) covering LFC values for 31,385 transcripts and 3846 proteins. Density plot was generated in R (v3.5.3).

***
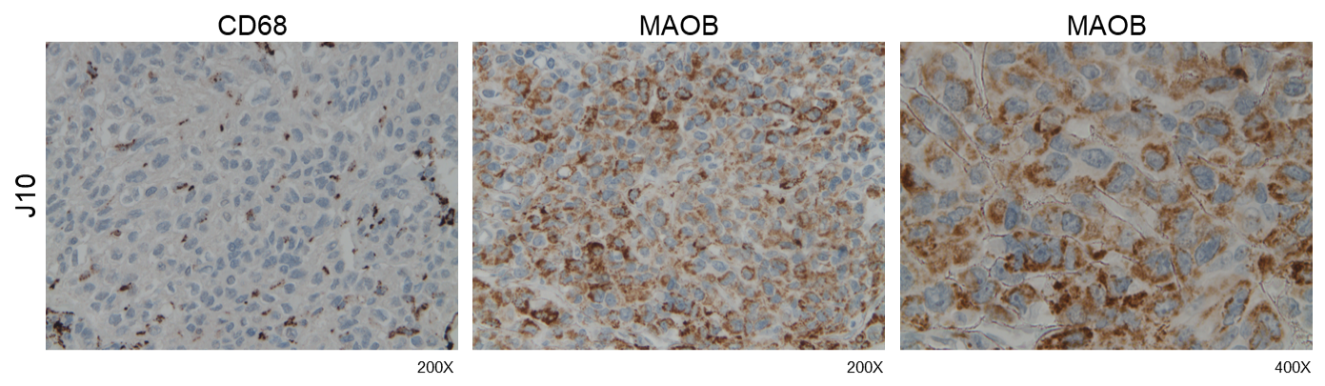
***

**Figure S2.** Expression of MAOB and CD68 in the grade III meningioma sample J10. Immunostaining for the common macrophage marker CD68 was performed to determine the extent of MAOB expression that may be derived from elements of the tumour stroma.


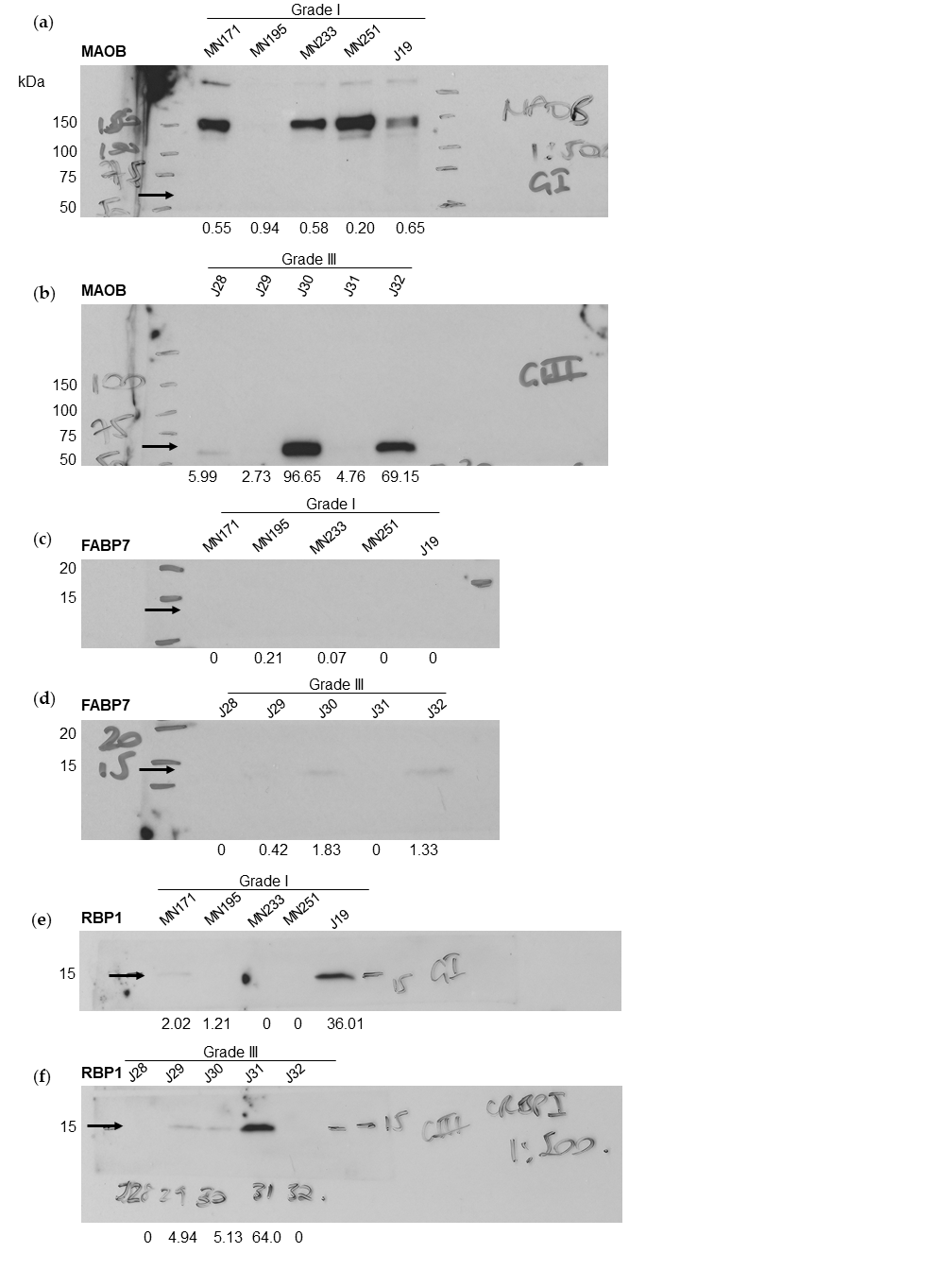


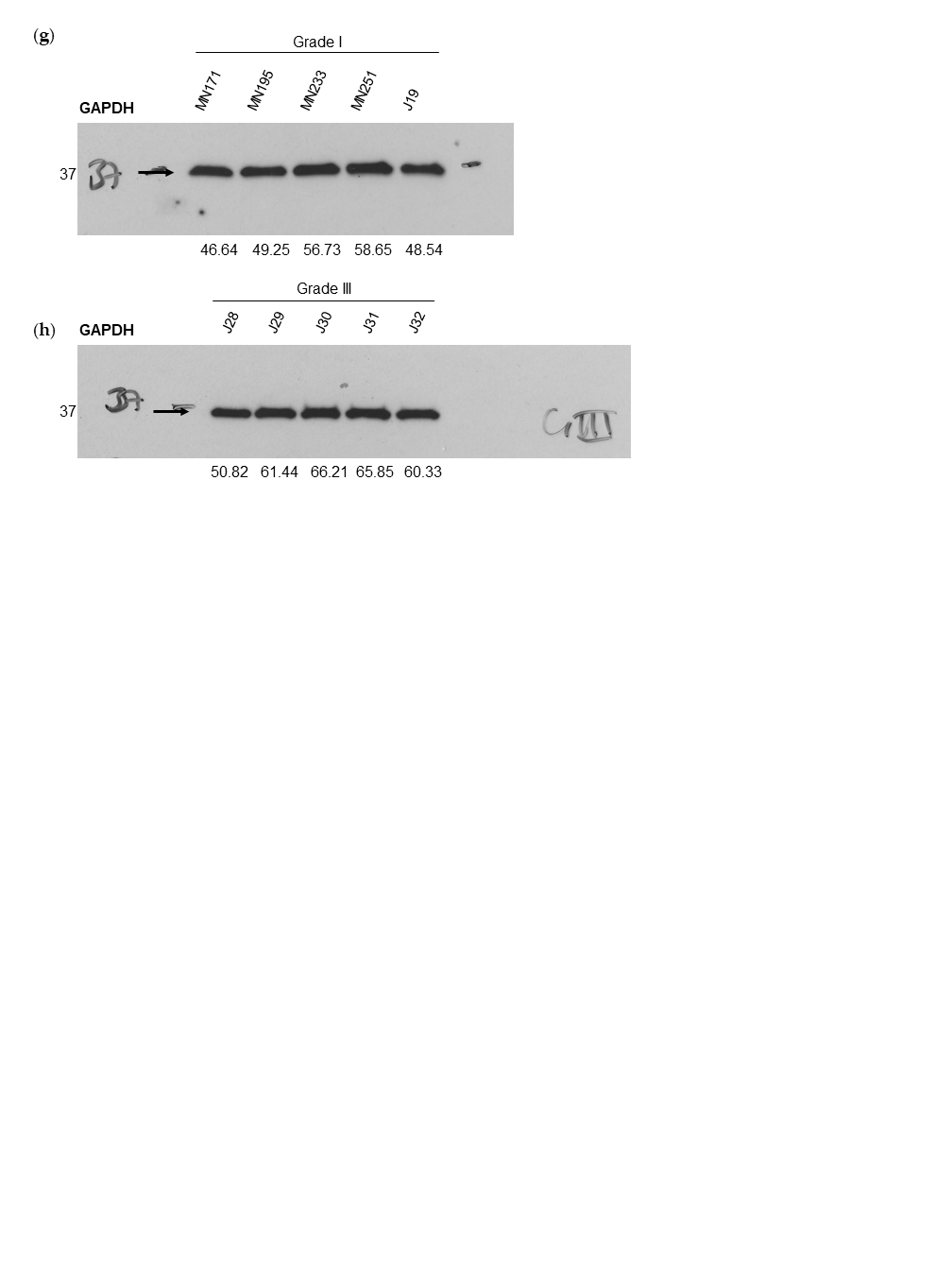


**Figure S3.** Uncropped Western blot gels for validation of proteins (Figure 4A) demonstrating concordantly increased expression in grade III vs. grade I meningioma. (**a**, **b**) MAOB expression (60 kDa) in grade I and grade III meningioma tissue lysates. Membranes were cut just below 50 kDa. (**c**, **d**) FABP7 expression (15 kDa) in grade I and grade III meningioma tissue lysates. Membranes were cut just above 20 kDa. (**e**, **f**) RBP1 expression (15 kDa) in grade I and grade III meningioma tissue lysates. Membranes were cut at 20 kDa. (**g**, **h**) GAPDH expression (37 kDa) in grade I and grade III meningioma tissue lysates. Membranes were cut just below 50 kDa and just above 20 kDa. Black arrows indicate expected expression for each protein.

**Table S2.** Primary antibody specifications used in Western blotting (WB) and immunohistochemistry (IHC).

| **Antigen** | **Antibody** | **WB dilution** | **IHC dilution** |
| --- | --- | --- | --- |
| MAOB | #sc-515354, Santa Cruz Biotechnology, Inc. | 1:500 |  |
| MAOB | #HPA002328, Sigma-Aldrich |  | 1:300 |
| FABP7 | #13347, Cell Signaling Technology^®^ | 1:1000 |  |
| FABP7 | #NBP1-88648, Novus Biologicals^®^ |  | 1:2000 |
| RBP1 | #sc-271208, Santa Cruz Biotechnology, Inc. | 1:500 | 1:75 |
| GAPDH | #MAB374, Merk Millepore | 1:50000 |  |
| CD68 | #M0876, Dako |  | 1:50 |
